# Common Variable Immune Deficiency and Pregnancy: Improving Outcomes Through Multidisciplinary Care

**DOI:** 10.3390/jcm15103810

**Published:** 2026-05-15

**Authors:** Fatemah Alyaqout, Michael Aw, Eisa Saleh, Derek Lee, Vanessa Polito, Michael Fein, Christos Tsoukas, Reza Alizadehfar, Genevieve Genest

**Affiliations:** 1 Division of Clinical Immunology and Allergy, McGill University Health Centre, 1650 Cedar Ave, Montreal, QC H3G 1A4, Canada; eisa.saleh@mail.mcgill.ca (E.S.); vanessa.polito@mcgill.ca (V.P.); michael.fein@mcgill.ca (M.F.); christos.tsoukas@mcgill.ca (C.T.); genevieve.genest@mcgill.ca (G.G.); 2 Division of Clinical Immunology and Allergy, Sheikh Jaber Al Ahmad Al Sabah Hospital, Ministry of Health Kuwait, Khalid Ben Abdul-Aziz Street, Kuwait 13018, Kuwait; 3 Division of Internal Medicine, Department of Medicine, McGill University Health Centre, 1001 Bd Décarie, Montreal, QC H4A 3J1, Canada; michael.aw@mail.mcgill.ca; 4 Department of Pharmacy, McGill University Health Centre, 1001 Bd Décarie, Montreal, QC H4A 3J1, Canada; derek.lee@muhc.mcgill.ca; 5 Division of Allergy Immunology and Dermatology, Montreal Children’s Hospital, McGill University Health Centre, 1001 Bd Décarie, Montreal, QC H4A 3J1, Canada; reza.alizadehfar.med@ssss.gouv.qc.ca

**Keywords:** common variable immune deficiency, pregnancy, immunoglobulin replacement therapy

## Abstract

**Background**: Pregnancy presents unique immunological and obstetrical challenges for women with Common Variable Immune Deficiency (CVID). No standardized guidelines currently exist to guide pregnancy management, as CVID is a rare diagnosis, with pregnancy outcomes limited to case reports and case series. Establishing a structured approach to care is important to optimize maternal and fetal outcomes. **Methods**: A narrative review of the literature with a structured search was performed to detail pregnancy outcomes in CVID and management strategies. A 10-year retrospective chart review of women with CVID who became pregnant while receiving care at the McGill University Health Centre between January 2015 and January 2025 was conducted to add to the existing clinical data. **Results**: Pregnancy outcomes were improved through pre-conception planning, regular serum Immunoglobulin G (IgG) monitoring, trimester-based immunoglobulin replacement dose adjustments, proactive management of autoimmune or infectious complications, and multidisciplinary care. Subcutaneous immunoglobulin may offer better flexibility and stability of IgG levels. **Conclusions**: In the available observational literature and our institutional experience, many patients with CVID have carried pregnancies to term with favorable maternal and neonatal outcomes when managed with IgRT and multidisciplinary coordination. We outline a stepwise multidisciplinary framework for clinicians caring for women with CVID who are planning or undergoing pregnancy, and we identify gaps in knowledge for future studies.

## 1. Introduction

Common Variable Immune Deficiency (CVID) is the most common symptomatic primary immune deficiency with an estimated prevalence of 1 in 25,000–50,000 individuals [1]. Contemporary registry data from the Latin American Society for Immunodeficiencies (LASID; *n* = 9307) confirms a growing population of women of reproductive age with CVID [2]. CVID is diagnosed in patients aged 4 years or older with (i) a marked decrease in serum IgG (typically ≥2 standard deviations below age-specific means) accompanied by reduced IgA and/or IgM, (ii) impaired antibody response to protein and/or polysaccharide vaccines, and (iii) the exclusion of secondary causes of hypogammaglobulinemia and other defined primary immunodeficiencies [3,4]. Advances in diagnosis and immunoglobulin replacement therapy (IgRT) have enabled many women with CVID to contemplate pregnancy [5]. However, the prognosis of pregnancy amongst CVID patients remains understudied. Indeed, gestation imposes distinctive immune, infectious, and obstetric demands that require tailored clinical decision-making in this population [6]. These include, but are not limited to, increased circulating volume, elevated Immunoglobulin G (IgG) catabolism, and IgG transplacental transfer to provide passive fetal immunity. These physiological changes necessitate individualized management strategies to optimize maternal and fetal outcomes.

No standardized guidelines exist either for the management of CVID during pregnancy or for other inborn errors of immunity [7]. Existing recommendations rely on case reports, case series, and a single multicenter retrospective cohort (PREPI) [5], with one recent single-center series of 33 women [8]. The clinical approach to intrapartum CVID care—including IgRT initiation and escalation, antimicrobial prophylaxis, vaccinations, and multidisciplinary care—remains unaddressed.

This gap in the literature reflects both the rarity of the condition and the historically limited reporting of pregnancy outcomes in this population. The lack of evidence-based guidance creates uncertainty for clinicians managing these complex pregnancies, potentially delaying care optimization and increasing adverse outcome risk. We performed a narrative review of the available literature on pregnancy outcomes of patients with CVID to identify a general approach to care. Our institutional case series is small (*n* = 4) and is presented as an illustrative experience rather than as a basis for generalizable inference. We retrospectively reviewed the outcomes of women with CVID followed at the Immune Deficiency Treatment Centre of the McGill University Health Centre between 2015 and 2025; this is a quaternary referral program for primary and secondary immunodeficiencies. We thus propose an algorithm for the management of such patients as a framework for further guidelines development.

## 2. Materials and Methods

### 2.1. Literature Review

We performed a comprehensive structured literature search to identify published studies on pregnancy management and outcomes in women with CVID. The search was executed on 8 April 2025 by an MUHC Health Sciences Library reference librarian. Databases searched: Ovid MEDLINE^®^ and Epub Ahead of Print, In-Process & Other Non-Indexed Citations and Daily (through 7 April 2025), and Embase—both accessed via the Ovid platform (Ovid Technologies/Wolters Kluwer Health, Philadelphia, PA, USA; underlying content: MEDLINE—National Library of Medicine, Bethesda, MD, USA; Embase—Elsevier, Amsterdam, Netherlands) Given the small and heterogeneous evidence base—predominantly individual case reports and small case series—the synthesis is presented as a narrative review with structured search rather than a formal systematic review. The full search strings, including controlled vocabulary and free-text terms, are provided in Supplementary File S1 for transparency.

#### 2.1.1. Electronic Database Search

A comprehensive search of MEDLINE (via **Ovid**) and Embase databases was performed for articles published between April 2005 and April 2025. The search was limited to articles published since 2005, as immunoglobulin replacement therapy preparations have been relatively standardized since then, and differ from earlier preparations. The search strategy combined controlled vocabulary (MeSH terms in MEDLINE) and free-text terms across three conceptual domains:

(1) CVID: “Common Variable Immunodeficiency,” “CVID,” “Hypogammaglobulinemia,” “Antibody Deficiency,” and related variations;
(2) Pregnancy: “Pregnancy,” “Pregnant,” “Gestation,” “Maternal,” “Antenatal,” “Prenatal,” “Perinatal,” “Obstetric,” “Delivery,” “Childbirth,” “Postpartum”;
(3) Management: “Disease Management,” “Treatment,” “Therapy,” “Care,” “Monitoring,” “Protocol,” “Guideline,” “Immunoglobulin,” “IVIG,” “SCIG,” “Prophylaxis,” “Antibiotic,” “Antimicrobial.”

#### 2.1.2. Study Selection Process

Titles and abstracts were screened by the lead reviewer (F.A.) with senior author oversight (G.G.). Studies were included if they (1) reported outcomes for women with confirmed CVID diagnosis who had completed pregnancy, (2) included maternal and/or fetal/neonatal outcomes with specific data reported, and (3) were full texts published in the English literature.

Studies were excluded if they were abstract-only publications, conference proceedings, editorials, opinion pieces, or narrative reviews without patient-level data; animal or in vitro studies; or studies describing other primary immunodeficiencies without specific CVID data. A full-text review of potentially eligible studies was conducted by the lead reviewer with senior author oversight (G.G.).

#### 2.1.3. Data Extraction and Quality Assessment

Data extraction was performed by the lead reviewer (F.A.) with verification by a senior co-author. Extracted data included (1) study characteristics (study ID, title, authors, journal, publication year, study type and design, data collection years, sample size), (2) population characteristics (number of CVID patients and pregnancies, diagnostic criteria used, maternal age, comorbidities, baseline IgG levels), (3) CVID-associated complications (ex: interstitial lung disease, gastrointestinal manifestations, autoimmune diseases), (4) intervention details (type of immunoglobulin replacement, dosing regimens, trimester-specific adjustments, use of antimicrobial prophylaxis), and (5) outcomes (maternal IgG levels during pregnancy, infection occurrence, obstetric complications, hospitalizations, live birth rates, gestational age at delivery, birth weight, neonatal IgG levels, neonatal complications, post-partum follow-up, adverse events related to IgRT). 

Quantitative synthesis (meta-analysis) was not attempted given the clinical, methodological, and statistical heterogeneity across the included studies—divergent outcome definitions (e.g., live-birth rate vs. term-delivery rate), inconsistent denominators (women vs. pregnancies), absence of comparator groups in case reports, and small individual sample sizes. A summary of study designs, sample sizes, and overall evidence strength is provided in Supplementary File S2.

### 2.2. Retrospective Chart Review

We retrospectively reviewed the management strategies as well as maternal and fetal outcomes for all patients with CVID who experienced pregnancy while receiving care at McGill University Health Centre’s IDTC clinic between January 2015 and January 2025. Eligible participants included pregnant women with a confirmed diagnosis of CVID according to established diagnostic criteria [3]: significantly reduced serum immunoglobulin levels (IgG, IgA, and/or IgM at ≥2 standard deviations below age-specific means), impaired antibody responses to vaccines, and exclusion of alternative causes of hypogammaglobulinemia. Patients with other documented immunodeficiencies were excluded from the analysis. Only patients with known pregnancy and neonatal outcomes were included. Ethics approval was obtained from the McGill University Health Centre Research Ethics Board (study number 2025-11526). Individual consent was waived for this study as it involved a chart review of de-identified clinical data with no direct patient contact.

#### Data Collection and Extraction

Data collected included the following: maternal demographics and CVID details (age, diagnostic criteria, baseline immunoglobulin levels, CVID-associated complications, genetic mutations); preconception counseling and planning details; pregnancy details (gravidity, parity, dates of conception and delivery, trimester-specific IgG levels, dosing adjustments of IgRT); infectious and autoimmune complications during pregnancy; obstetric complications; delivery details (mode, gestational age, complications); neonatal outcomes (birth weight, neonatal IgG levels, cord blood findings, NICU admission, neonatal complications); post-partum maternal course; post-partum IgRT management. Data extraction was performed by the lead investigator (F.A.) with verification by a second reviewer for completeness and accuracy.

## 3. Results

### 3.1. Narrative Review

#### 3.1.1. Immunoglobulin Replacement Therapy

Across the included studies [5,9,10,11,12,13,14], immunoglobulin replacement was consistently described as the central intervention during pregnancy. Both intravenous (IVIG) and subcutaneous (SCIG) routes were represented in the literature. Intravenous administration was the more commonly described route, with most cohorts reporting upward dose titration over the course of gestation—most often in the third trimester—to keep maternal IgG within target [5,14]. A minority of patients received facilitated subcutaneous immunoglobulin (fSCIG); the available reports describe acceptable maternal tolerance and no excess of neonatal adverse events [15]. Overall, no differences in maternal or neonatal infection rates were reported between IVIG, SCIG, and fSCIG users. Monitoring frequency varied across studies, with most employing monthly or trimester-based IgG measurements [14,16]. Studies that employed regular monitoring reported fewer infectious complications during pregnancy [12,16].

#### 3.1.2. Antimicrobial Prophylaxis

**Figure 1:**
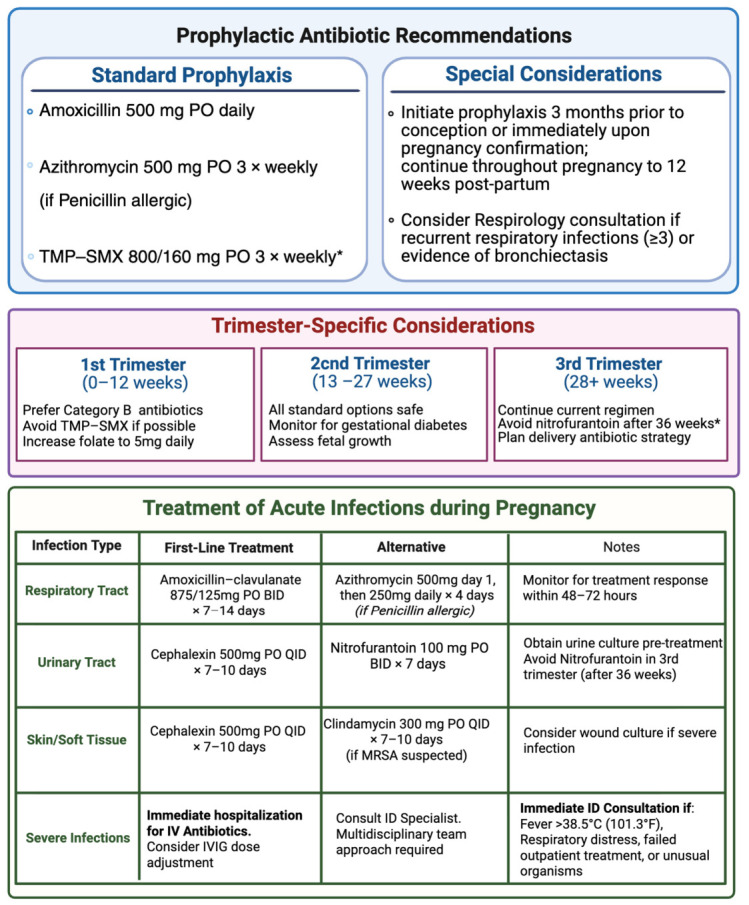
Antibiotic recommendations in CVID pregnancy. Created in BioRender (Science Suite Inc., Toronto, ON, Canada; web application, accessed May 2026; https://www.biorender.com). Al yaqout, F. (2026) https://BioRender.com/dp6dmyy. * SMX/TMP: Sulfamethoxazole 800 mg/Trimethoprim 160 mg—avoid in 1st trimester due to neural tube defect risk.

Antimicrobial prophylaxis was typically used in patients with recurrent infections or underlying bronchiectasis [5,14]. In the PREPI study, antimicrobial prophylaxis was associated with higher live birth rates among women with primary immunodeficiency (82% vs. 63% without optimal prophylaxis) [5]. Common prophylactic agents included azithromycin and trimethoprim–sulfamethoxazole [4]. Prophylaxis was generally initiated during the second trimester and was not associated with major adverse events [17]. No standardized protocols for prophylaxis initiation or duration were identified across studies (Figure 1).

#### 3.1.3. Maternal Outcomes

Maternal infection rates during pregnancy were low among women who maintained adequate IgG levels. Mild respiratory infections were the most frequently reported complications. No maternal mortalities or severe sepsis events were reported. Obstetric complications, including preeclampsia and preterm labor, were reported in some cases but were not consistently linked to CVID [11].

#### 3.1.4. Neonatal Outcomes

Overall, term live birth rates were comparable to the general population across studies. Neonatal outcomes were largely favorable, with normal birth weights predominating. Cord blood IgG levels were within or near normal reference ranges when reported [5,12,14]. NICU admissions were infrequent and largely unrelated to CVID or maternal immunodeficiency management. Of note, Mallart et al. identified a history of severe maternal infections as a risk factor for poor obstetrical and neonatal outcomes compared to the general population (aOR 0.33, 95% CI 0.12–0.90, *p* = 0.031) [5].

### 3.2. Retrospective Review

In total, four patients with CVID who completed a pregnancy while under the care of the IDTC clinic were identified from a retrospective chart review. The four cases below are presented as illustrative examples of contemporary management at a quaternary referral center and are not intended to support generalizable inference.

Case 1: A 35-year-old (G7P1A4) presented with recurrent miscarriages prior to her CVID diagnosis. Her first pregnancy (age 30) was a term pregnancy complicated by fetal intra-uterine growth restriction (IUGR) and maternal thrombocytopenia. Her son was born by C-section at 2900 g (10th percentile), with pathologic evidence of placental vascular malperfusion. She then had two first-trimester miscarriages and two therapeutic abortions at 28 and 20 weeks’ gestation (GW) for multiple congenital malformations. Her partner was found to have a pathogenic FLT-4 mutation, and the couple underwent IVF for pre-implantation genetic screening for monogenic disorders (PGT-M). She subsequently miscarried a euploid non-affected embryo and failed two euploid, unaffected embryo transfers. During her son’s integration to daycare, she developed recurrent sinopulmonary infections. Workup revealed low IgG (3.37 g/L), low IgA levels (0.33 g/L), low/absent serum IgG titers to diphtheria, tetanus, *Haemophilus influenzae*, and *Streptococcus pneumoniae*, as well as mild splenomegaly and thrombocytopenia. Her seventh pregnancy was unplanned. Hizentra^®^ 6 g/week (0.4 g/kg), low-dose aspirin, and Tinzaparin 4500 units daily were started at GW6. She was treated for an episode of sinusitis at GW10 and Hizentra^®^ was increased to 8 g/week (0.6 g/kg). At GW28, Hizentra^®^ was increased to 10 g/week (0.8 g/kg) to maintain IgG levels of 10 g/L and she received influenza, tetanus–diphtheria–acellular pertussis (Tdap), and respiratory syncytial virus (RSV) vaccines. She had an uncomplicated pregnancy and a term vaginal delivery of a healthy male (3040 g). Post-partum, Hizentra^®^ was decreased to 6 g/week, and she breastfed without complications.

Case 2: A 37-year-old (G2P2) was diagnosed with CVID after her first pregnancy, which was complicated by pre-term premature rupture of membranes at 34 weeks. She delivered a small-for-gestation-age boy (1810 g, 12th percentile), who required 2-week hospitalization in the neonatal intensive care unit (NICU). Post-partum, the patient developed an empyema requiring IV antibiotics and surgical drainage, subsequently presenting with recurrent sinopulmonary infections. Investigations revealed IgG 4.07 g/L, IgA < 0.05 g/L, and poor pneumococcal vaccine response. Genetic testing demonstrated a likely pathogenic variant in Tumor Necrosis Factor Superfamily Member 12 (TNFSF12) and Uracil DNA Glycosylase (UNG). She was maintained on Hizentra^®^ 4 g/week (0.3 g/kg) weekly and remained infection-free with stable IgG levels of 10 g/L for over 6 months prior to her second pregnancy. Influenza vaccination was given at GW 16. By GW 23, IgG levels decreased to 7.8 g/L and Hizentra^®^ was increased to 6 g weekly (0.6 g/kg). Pregnancy was complicated by immune thrombocytopenia at GW 28 with good response to steroids. She required an urgent C-section at GW 34 for pre-term labor and thrombocytopenia, delivering a healthy boy who did not require NICU admission. The infant did not have any infectious complications.

Case 3: A 26-year-old (G3P1A2) was diagnosed with CVID at age 5 (heterozygous Tumor Necrosis Factor Receptor Superfamily Member 13B (TNFRSF13B/TACI) mutation, liver cirrhosis (glycogen storage disease), and Fanconi syndrome). Her first two pregnancies were miscarriages (age 17 and 22 with different partners; she was not compliant with Hizentra^®^ and had recurrent infections at the time). Her third pregnancy was unplanned while on Hizentra^®^ 6 g weekly (0.4 g/kg) with a stable IgG of 9.67 g/L. During pregnancy, she developed worsening proteinuria; Hizentra^®^ was increased to 8 g/week at GW 10, 10 g at GW 25, and ultimately 12 g weekly at GW 32; IgG peaked at 7.29 g/L despite dose escalation. She received appropriate vaccinations during pregnancy (Tdap at GW 24, influenza at GW 25, and COVID-19 at GW 28), but contracted COVID-19 at GW 31.5 and developed pre-term labor (PTL). She was hospitalized for 48 h and treated with tocolytics, sotrovimab, amoxicillin, and betamethasone for fetal lung maturity with resolution. At GW 34, she went into PTL and had an uneventful vaginal delivery of a 3680 g baby girl. Her daughter was hospitalized in the NICU for 3 weeks for ongoing hypoglycemia. There were no neonatal infectious complications.

Case 4: A 30-year-old woman (G2P2) with long-standing CVID and stable SCIG therapy experienced two uncomplicated term pregnancies without infectious or neonatal complications. She remained clinically stable for several years on Hizentra^®^ 6 g/week before conceiving her first planned pregnancy at age 28, which proceeded uneventfully without dose adjustments and IgG levels > 10 g/L. She delivered a term boy (3410 g) without complications. In her second pregnancy at age 30, her IgG dropped to 7.26 g/L at GW 28, prompting an increase in Hizentra^®^ to 9 g/week. She again had an uncomplicated term delivery of a 3570 g boy.

## 4. Discussion

**Figure 2:**
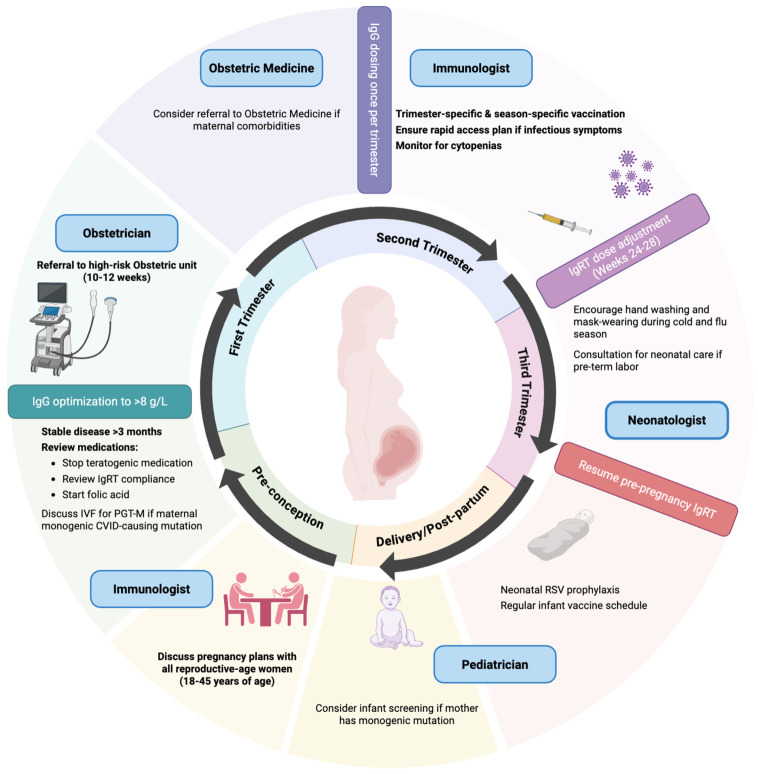
Management of CVID during pregnancy. Created in BioRender. Al yaqout, F. (2026) https://BioRender.com/jrp5h3a.

**Figure 3:**
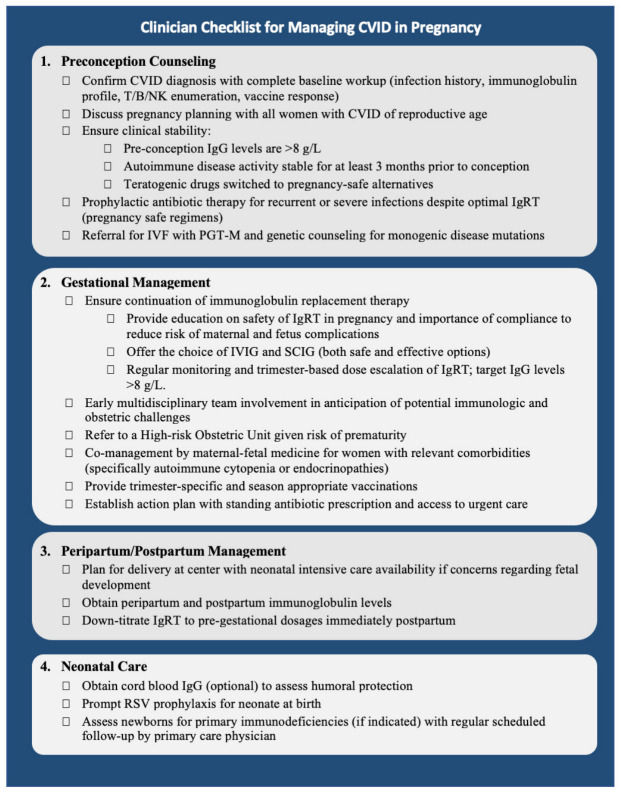
Pregnancy management checklist for patients with CVID. Figure created using Microsoft Word (Microsoft Corporation, Redmond, WA, USA; version 16.108.3/Microsoft 365).

Strength of evidence. The body of evidence reviewed here consists predominantly of small case series and individual case reports, with two retrospective cohorts (PREPI, Mallart 2023 [5]; Kralickova 2015 [11]) and one contemporary single-center cohort (Kalkan 2026, *n* = 33 [8]) providing the highest-quality observational data. A summary of study designs, sample sizes, and overall evidence strength is provided in Supplementary File S2. Throughout the discussion below, the findings derived from cohort and registry data are weighted more heavily than findings from individual case reports, and recommendations in the proposed management algorithm are explicitly labeled as consensus- and experience-based unless directly supported by cohort-level data, in which case the contributing study is named in-line. A functional maternal immune system is required for a healthy pregnancy. Indeed, maternal immune cells are involved in embryo recognition, implantation, placenta formation, and fetal tolerance throughout gestation [18]. Importantly, the maternal transplacental transfer of IgG antibodies provides a passive humoral protection for the first 3–6 months of neonatal life [18]. Dysregulation of the maternal immune system, such as in CVID, increases the risk of gestational complications and post-partum infections [5,11]. Over the past five years, we have observed an increasing number of women with CVID contemplating pregnancy within our clinic population. Based on our review of the literature and institutional clinical experience, we have identified several key components in the clinical management of such patients and propose these as foundational elements for multidisciplinary care (Figure 2). A practical bedside checklist summarizing the four management domains—preconception counseling, gestational management, peripartum/post-partum care, and neonatal care—is provided in Figure 3.

This checklist is intended as a clinical aid; recommendations are consensus- and experience-based and should be applied with individualization to the patient and in coordination with maternal–fetal medicine.

### 4.1. Preconception Counseling

- Confirm CVID diagnosis with complete baseline workup (infection history, immunoglobulin profile, T/B/NK enumeration, vaccine response);
- Discuss pregnancy planning with all women with CVID of reproductive age;
- Ensure clinical stability: pre-conception IgG > 8 g/L; autoimmune disease activity stable for ≥3 months; teratogenic medications switched to pregnancy-safe alternatives;
- Initiate prophylactic antibiotics for recurrent or severe infections despite optimal IgRT (pregnancy-safe regimens);
- Refer for IVF with PGT-M and genetic counseling when monogenic, CVID-causing mutations are identified.

### 4.2. Gestational Management

- Continue IgRT throughout pregnancy; offer choice of IVIG or SCIG (both safe and effective);
- Monitor IgG monthly, with trimester-based dose escalation targeting IgG > 8 g/L;
- Engage the multidisciplinary team (immunology, maternal-fetal medicine, obstetrics) early;
- Refer to a high-risk obstetric unit given the risk of prematurity;
- Co-manage with maternal-fetal medicine for relevant comorbidities (autoimmune cytopenia, endocrinopathies);
- Provide trimester-specific and season-appropriate vaccinations;
- Establish an action plan with standing antibiotic prescription and access to urgent care.

### 4.3. Peripartum/Post-Partum Management

- Plan delivery at a center with neonatal intensive care availability when fetal-development concerns are present;
- Obtain peripartum and post-partum maternal IgG levels;
- Down-titrate IgRT to pre-gestational dosing in the immediate post-partum period.

### 4.4. Neonatal Care

- Obtain cord-blood IgG (optional) to assess humoral protection;
- Consider neonatal RSV prophylaxis at birth (e.g., maternal RSVpreF vaccination history, nirsevimab);
- Assess newborns for primary immunodeficiencies where indicated, with scheduled follow-up by the primary care physician.

Pregnancy should be discussed with all CVID patients of reproductive age. In patients with IgG levels < 8 g/L, active infection, recent recurrent infections, or active autoimmune disease, pregnancy should be delayed until disease stability is achieved at least 3–6 months prior to conception attempts. Medications such as prophylactic antibiotics need to be tailored for pregnancy (Figure 2) and patients with suspected penicillin allergy should be assessed for allergy de-labeling prior to pregnancy. For patients with co-existing auto-immune disease, teratogenic medications must be replaced with pregnancy-safe alternatives prior to conception attempts. In patients with known monogenic disease mutations, risk of transmission to the fetus should be discussed. A referral to a fertility specialist for in vitro fertilization (IVF) with Preimplantation Genetic Testing for Monogenic Disorders (PGT-M) or to a geneticist for peri-natal screening should be offered.

(1)
**Immunoglobulin replacement therapy:**

IgRT is safe, well-tolerated, and a cornerstone of CVID management during pregnancy [19]. Compliance with IgRT must be reviewed at each preconceptual and pregnancy visit. SCIG has been shown to be as safe and effective as IVIG in the treatment of CVID patients [1,15]. SCIG must be given special consideration in pregnancy due to the flexibility of administration and more consistent serum IgG levels [20]. Alternatively, fSCIG (HyQvia^®^) enables higher-volume delivery with less frequent dosing compared to conventional SCIG. This reduces injection burden and should be considered [10]. Due to increased circulating volume, increased IgG catabolism during pregnancy [11], and IgG transplacental transfer in the third trimester [21,22], serum IgG should be performed once per trimester (or more frequently if the patient presents with infections) with adjustment to maintain serum IgG levels > 8 g/L. In our experience, dose escalation of 20–50% was typically required during the second and third trimesters, consistent with the available literature (Table 1). Of note, two of our patients had multiple miscarriages prior to IgRT compliance or CVID diagnosis. Following the initiation of IgRT, the successful birth of two healthy children was achieved, an observation suggestive of a potential benefit of IgRT in this setting. Similarly, a retrospective (*n* = 50) Czech study found higher rates of pre-term labor, small gestational weight, and stillbirths compared to the general population [11]. The number of unsuccessful pregnancies was greater among those who did not receive IgRT. A recent Spanish multicenter study (*n* = 212) confirmed the broadly comparable infection-prevention performance of IVIG and SCIG for CVID, providing further real-world support for individualized modality selection [23]. Overall, the available observational data—including the recent single-center cohort of 33 women by Kalkan et al. [8]—are consistent with, but do not establish, a benefit of optimized IgRT on reproductive outcomes [19].

(2)
**Multidisciplinary management:**

CVID pregnancies should be considered high-risk and managed as such. A normal immune response to the semi-allogenic embryo/fetus is required to promote tolerance to the conceptus and ensure a complication-free pregnancy [24]. Immune dysregulation, as is the case in CVID, may lead to an increase in maternal and fetal complications as well as post-partum infections [5,11]. Indeed, a retrospective study found higher rates of pre-term labor, small gestational weight, and stillbirths in mothers with CVID compared to the general population [11]. We recommend that all CVID patients be referred to a high-risk obstetric unit; in CVID patients with autoimmune comorbidities (specifically cytopenia or endocrinopathies), co-management by Obstetric Medicine should also be considered. Since CVID patients are at high risk of infection, a rapid access plan for care must be available should any infectious symptoms arise. Severe maternal infections are associated with fetal demise in primary immunodeficiency, and timely management is crucial to reduce adverse pregnancy outcomes [5,25].

(3)
**Vaccinations:**

All pregnant CVID patients should receive trimester-specific and season-appropriate vaccinations. While IgRT contains protective antibodies to viruses to which the population has developed herd immunity, it will not protect against emerging viral strains (COVID-19, influenza, RSV). Maternal bivalent prefusion F (RSVpreF) vaccination is now supported by both controlled trial data (MATISSE) [26] and real-world UK effectiveness data showing a 58–72% reduction in infant RSV hospitalization [27]. Additionally, despite RSV vaccination, CVID mothers may not be able to mount a protective antibody response. We recommend neonates receive RSV prophylaxis (e.g., palivizumab, nirsevimab) at birth.

### 4.5. Limitations and Future Directions

Our study has several important limitations. The retrospective case series from a single quaternary center limits generalizability to diverse CVID populations. Specifically, our cases represent a selected population with access to specialized immunodeficiency care, which may not reflect the experience of women with CVID managed in community settings. Furthermore, the integration of a narrative review with a case series, while strengthening the clinical context, also creates methodological challenges in distinguishing findings based on robust evidence and local expert opinion.

The narrative review’s restriction to English-language publications and the 2005–2025 search period, while justified by IgRT standardization, may have excluded relevant non-English literature and important historical cases. The heterogeneity of reporting across studies (different outcome definitions, follow-up durations, and data collection methods) prevented meta-analytic synthesis. Individual study sample sizes were small, mostly case reports and small case series, which limits the strength of evidence regarding specific management approaches. The literature scoping was not registered prospectively (e.g., with PROSPERO) and a formal risk-of-bias scoring rubric (such as a formal risk-of-bias scoring rubric (such as the Newcastle–Ottawa Scale [28]) was not applied; instead, an overall narrative judgment of evidence strength is provided in Supplementary File S2. Reframing the article as a narrative review rather than a formal systematic review reflects these methodological constraints transparently.

Despite these limitations, this work provides a comprehensive synthesis to date of CVID pregnancy management and serves as a foundation for future research. Several important directions for future investigation are identified: (1) Prospective, multi-center studies documenting standardized IgG targets, as well as outcomes in larger CVID pregnancy cohorts. (2) Registry-based studies capturing contemporary practice patterns and long-term outcomes. (3) Mechanistic studies examining the physiologic basis for increased IgG catabolism in CVID pregnancies and factors predicting which patients will require substantial dose escalation. (4) Comparative studies evaluating outcomes between IVIG, SCIG, and fSCIG in pregnancy. (5) Health services research examining models of multidisciplinary care coordination that optimize outcomes and minimize adverse events.

## 5. Conclusions

In the available observational literature and our institutional experience, women with CVID have carried pregnancies to term with favorable maternal and neonatal outcomes when managed with individualized IgRT protocols, regular immunological surveillance, multidisciplinary coordination, and proactive infection prevention. This narrative synthesis brings together the contemporary evidence on CVID pregnancy management—including the most recent single-center cohort by Kalkan et al. (2026) [8], the PREPI multicenter cohort [5], and our four-case institutional series—and proposes a consensus- and experience-based management algorithm with explicit evidence tiers, accompanied by a practical clinical checklist for multidisciplinary teams. Until prospective multi-center studies and international registries provide higher-quality evidence, the principles outlined here can support clinicians caring for this growing patient population. Key gaps for future investigation include prospective IgG-target outcome studies; registry-based capture of contemporary practice; mechanistic work on increased IgG catabolism in CVID pregnancies; comparative studies of IVIG, SCIG, and fSCIG; and health-services research on multidisciplinary care coordination.

## Figures and Tables

**Table 1:** Summary of included studies on CVID and pregnancy.

First Author/Year	Study Design Country	No. of Patients	Inclusion Criteria	Type of Immunoglobulin	Outcome	Recommendations
Manson 2012 [9]	Case Report Retrospective Year of study not specified United Kingdom	1 patient 2 pregnancies 2 live births	34-year-old patient diagnosed with granulomatous CVID 24 GW of second pregnancy (low Ig levels, impaired vaccine response to tetanus, recurrent respiratory tract infections since childhood)	IVIG (type not specified), started 15 g/month (0.27 g/kg) at 26 GW, increased to 22 g (0.4 g/kg) at 32 weeks and to 30 g (0.54 g/kg) at 37 GW	The patient had an uncomplicated term pregnancy prior to CVID diagnosis Second pregnancy complicated by deep vein thrombosis and pseudomonas bacteremia at 24 GW. IVIG started at 26 GW. Cholestasis of pregnancy at 37 weeks. Urgent C-section for placental abruption, post-partum hemorrhage requiring uterine artery embolization Healthy male infant	Consider CVID pregnancies high-risk Increase IgRT dose to maintain stable IgG levels
Danieli 2012 [10]	Case Report Retrospective January 2011–February 2012 Italy	1 patient 2 pregnancies 1 live birth 1 miscarriage	42-year-old patient with CVID (longstanding recurrent upper respiratory tract infections, pan-hypogammaglobulinemia, impaired tetanus vaccine response)	10% liquid IVIG; then, Privigen^®^ (0.6 g/kg/month)	Patient had one spontaneous miscarriage at 8 weeks while on IVIG Post-miscarriage: recurrent infusion reaction to IVIG and discontinuation of therapy Second pregnancy: Alternative IgRT (Privigen^®^ 10%; 0.6 g/kg/month) started at 18 GW with premedication and slow infusion rate; IgG levels maintained > 10 mg/dL Term; healthy boy (40 GW), breast fed Privigen decreased to 0.4 g/month post-partum	Privigen^®^ preparation is well tolerated in patients with adverse reactions to IVIG IVIG is necessary to prevent maternal/fetal infections Dose increased must be considered in pregnancy
Kralickova 2015 [11]	Cohort study Retrospective Year of study not specified Czech Republic	50 patients 115 pregnancies 88 live births (92 children, 2 sets of twins) 12 miscarriages 3 stillbirths 11 pregnancy terminations (9 by choice, 2 for congenital anomalies) 1 ectopic pregnancy	Group A (*n* = 85): pregnancies occurring before CVID manifestations Group B (*n* = 14): pregnancies occurring after first CVID symptoms but before IgRT Group C (*n* = 16): pregnancies occurring in women with established CVID and on IgRT	Not specified	Higher rates of preterm labor, eclampsia and pre-eclampsia, low birth weight offspring, and stillbirths in CVID patients compared to the general population Higher rates of antibiotic use during pregnancy in Group B Higher rates of antibiotic administration to offspring 0–12 months in Group B IVIG and scIg were equally effective in preventing infectious complications	Consider CVID pregnancies high-risk pregnancies Continue IgRT during pregnancy Increase IgRT dose to maintain stable trough IgG levels
Marasco 2017 [12]	Case Report Retrospective 2008–2012 Italy	1 patient 2 pregnancies 2 live births	36-year-old patient diagnosed with CVID after second pregnancy (recurrent respiratory infections, pan-hypogammaglobulinemia, impaired tetanus vaccine response)	Hizentra^®^ 20% 8 g/week (0.4 g/kg/month) started at CVID diagnosis, increased to 10 g/week at 28 weeks until delivery	First pregnancy complicated by ITP prior to CVID diagnosis Later diagnosis of CVID (recurrent respiratory infections, low immunoglobulin levels, impaired tetanus vaccine response) Second pregnancy on SCIG uncomplicated, term birth; healthy girl	Increase IgRT dose during 3rd trimester to maintain stable IgG levels SCIG is safe during pregnancy
Sheikhbahaei 2018 [13]	Case Series Retrospective Year of study not specified Iran	3 patients 3 live births	9 patients with PID 3 pregnant patients with CVID	IgRT not specified	One patient on 0.4 g/kg/3 week IVIG had ITP and increased to 0.8 g/kg/3 week in 2nd trimester. Term; healthy child Patient with CVID and autoimmune hepatitis, off IgRT (allergy), stopped Cellcept^®^ and prednisone at diagnosis of pregnancy (with normalization of liver enzymes). Eclampsia at 24 weeks, conservatively managed with delivery at 38 GW One patient with CVID had previous pregnancy prior to diagnosis, which was complicated by recurrent pneumonia and severe dyspnea throughout pregnancy	Untreated CVID can increase risk of adverse maternal and fetal outcomes (infections, ITP, eclampsia) Increase IgRT dose during pregnancy
Egawa 2019 [14]	Case Series Retrospective January 2007–December 2016 Japan	4 patients 9 pregnancies 8 live births 1 miscarriage	Pregnant patients with CVID on IgRT	‘10% liquid IVIG’ (Japan Blood Products Organization) and/or Hizentra 20% Target IgG Target IgG level ≥ 10 mg/dL	One patient had a 12-week miscarriage and 3 healthy pregnancies Patients with IgG levels <10 mg/dL had more recurrent infections No other adverse obstetrical or perinatal adverse events	Increase IgG dose during pregnancy IVIG: shorten interval to every 2 weeks from 28–35 weeks and weekly from 36 weeks to delivery scIg: shorten dosing interval to twice per week after 28 weeks until delivery, add IVIG in patients not maintaining target IgG levels
Mallart 2023 [5]	Retrospective observational, monocentric study 1966–2022 France	51 patients with ‘primary antibody deficiency’ 119 pregnancies 82 live births 27 miscarriages 2 ectopic pregnancies 2 pregnancies losses > 20 weeks 7 voluntary pregnancy terminations, 1 medical termination of pregnancy	Patients with ‘primary antibody deficiency’ 31 patients became pregnant while on IgRT	Not specified	Better outcomes in pregnancies treated with IgRT 68/78 (87%) term birth 10/78 (17%) pre-term birth (32–37 weeks 7/78 (3%) extreme pre-term birth (<32 weeks) 16% low birth weight (<10th percentile) 7/69 (10%) infection during neonatal period	Trimester-specific and season-specific vaccination Multidisciplinary care Discontinue teratogenic medication and substitute when possible Increase IgRT during pregnancy if IgG levels are ‘low’

Abbreviations: CVID, common variable immunodeficiency; IgRT, immunoglobulin replacement therapy; IVIG, intravenous immunoglobulin; SCIG, subcutaneous immunoglobulin; GW, gestational week; ITP, immune thrombocytopenia.

## Data Availability

The original contributions presented in this study are included in the article/supplementary material. Further inquiries can be directed to the corresponding author.

## Supplementary Materials

The following supporting information can be downloaded at: https://www.mdpi.com/article/10.3390/jcm15103810/s1, File S1: Full database search strategy (Ovid MEDLINE and Ovid Embase), including controlled vocabulary, free-text terms, and Boolean operators. File S2: Quality assessment of included studies, performed independently by three reviewers using JBI Critical Appraisal Checklists (case reports and case series) and the Newcastle-Ottawa Scale (retrospective observational studies).
