# Supplementary material for: Common Variable Immune Deficiency and Pregnancy: Improving Outcomes Through Multidisciplinary Care
Source: J Clin Med. 2026 May 15;15(10):3810. doi: 10.3390/jcm15103810 (PMC13206869; doi:10.3390/jcm15103810)
Supplement: Supplementary file 1 [file jcm-15-03810-s001.zip › File_S1_SearchStrategy.pdf]

Supplementary File S1: Structured Literature Search Strategy

Manuscript: Common Variable Immune Deficiency and Pregnancy: Improving Outcomes Through Multidisciplinary Care.

Article type: Narrative review with structured literature search.

Purpose of this supplement: This document is provided for transparency — narrative reviews do not require formal PRISMA-style search reporting. It is included to allow interested readers to assess the breadth and rigour of the underlying literature scoping that informed the review.

1. Research Question and PICO Framework

The structured search was guided by a predefined PICO framework focused on identifying optimal management strategies for pregnant women with Common Variable Immunodeficiency (CVID) to ensure favorable maternal and fetal outcomes.

Population: Women with confirmed CVID who were pregnant, planning pregnancy, or had completed pregnancy.

Intervention: Any management approach during pregnancy, including immunoglobulin replacement therapy (IVIG, SCIG, or fSCIG), antimicrobial prophylaxis, vaccination, or structured monitoring protocols.

Comparison: Different management strategies (e.g., pre-pregnancy vs. during pregnancy approaches; IgRT-replete vs. IgRT-naïve), or standard care.

Outcomes: Maternal outcomes (infection rates, CVID-related complications), pregnancy outcomes (miscarriage, preterm birth, obstetric complications), and fetal/neonatal outcomes (birth weight, neonatal IgG levels, NICU admission, congenital findings).

2. Databases, Platform, and Execution

Databases: Ovid MEDLINE® and Epub Ahead of Print, In-Process & Other Non-Indexed Citations and Daily (through 7 April 2025); Embase (Ovid platform).

Search executed: 8 April 2025 by Ibtisam Mahmoud, McGill University Health Centre Health Sciences Library (MGH Medical Library) reference librarian.

Date limits: Articles published between April 2005 and April 2025. The 2005 cut-off was selected because contemporary immunoglobulin replacement formulations were standardised by this time, making earlier literature less representative of current practice.

Language: English. Non-English studies were considered if an accessible English translation was available.

Reporting standards: The literature scoping was structured according to structured reporting principles to support transparency, though the final manuscript is presented as a narrative review rather than a formal systematic review or meta-analysis.

3. Ovid MEDLINE Search Strategy

The structured strategy below was executed in Ovid MEDLINE on 8 April 2025 and combined controlled vocabulary (MeSH) with free-text terms across three concept domains (CVID, pregnancy, management).

#	Search	Results
**1**	Common Variable Immunodeficiency/	2,724
**2**	(("CVID" or (common or acquired or late-onset or primary)) adj3 (immunodeficienc* or immune-deficienc* or hypogammaglobulinemia* or immunoglobulin* or agammaglobulinemia*)).tw,kf.	41,515
**3**	1 OR 2	41,886
**4**	exp Pregnancy/	1,056,125
**5**	(pregnanc* or gestat* or gravidit* or childbear* or child bear*).tw,kf.	714,356
**6**	4 OR 5	1,254,889
**7**	3 AND 6	1,432
**8**	(manag* or admin* or treat* or therap*).tw,kf.	11,064,749
**9**	ad.fs. (administration & dosage subheading)	1,555,955
**10**	8 OR 9	11,647,900
**11**	7 AND 10	712
**12**	limit 11 to last 20 years	236

4. Embase (Ovid) — Translated Search Strategy

A parallel Embase (Ovid) search was performed using equivalent Emtree controlled vocabulary and free-text terms across the same three concept domains. The translated strategy follows the same Boolean structure as the MEDLINE search above:

Population (CVID):

```
'common variable immunodeficiency'/exp OR ((CVID OR ((common OR acquired OR
"late-onset" OR primary) NEAR/3 (immunodeficienc* OR "immune-deficienc*" OR
hypogammaglobulinaemi* OR hypogammaglobulinemi* OR immunoglobulin* OR
agammaglobulinaemi* OR agammaglobulinemi*))):ti,ab,kw
```

Setting (Pregnancy):

```
'pregnancy'/exp OR (pregnanc* OR gestat* OR gravidit* OR childbear* OR "child
bear*"):ti,ab,kw
```

Management/Intervention:

```
'disease management'/exp OR 'drug therapy'/exp OR (manag* OR admin* OR treat*
OR therap*):ti,ab,kw
```

Combined: (Population) AND (Pregnancy) AND (Management). Filters applied: English language; Publication years 2005–2025. Total Embase records retrieved: 500.

The full Embase RIS export and the librarian's annotated search history are available on request.

5. PubMed-equivalent Search String (for Reproducibility)

For reproducibility outside the Ovid platform, the following PubMed-translatable string represents the same conceptual search:

Population (CVID):

```
"Common Variable Immunodeficiency"[Mesh] OR "Common Variable
Immunodeficiency"[tiab] OR "Common Variable Immune Deficiency"[tiab] OR
CVID[tiab] OR "Hypogammaglobulinemia"[tiab] OR "Antibody Deficiency"[tiab]
```

Setting (Pregnancy):

```
"Pregnancy"[Mesh] OR "Pregnant"[tiab] OR "Pregnancy"[tiab] OR
"Gestation"[tiab] OR "Gestational"[tiab] OR "Maternal"[tiab] OR
"Maternity"[tiab] OR "Antenatal"[tiab] OR "Prenatal"[tiab] OR
"Perinatal"[tiab] OR "Obstetric"[tiab] OR "Labor"[tiab] OR "Delivery"[tiab] OR
"Childbirth"[tiab] OR "Postpartum"[tiab] OR "Postnatal"[tiab] OR
"Breastfeeding"[Mesh] OR "Breastfeeding"[tiab] OR "Breast Feeding"[tiab] OR
"Lactation"[tiab]
```

Management:

```
"Disease Management"[Mesh] OR "Management"[tiab] OR "Treatment"[tiab] OR
"Therapy"[tiab] OR "Care"[tiab] OR "Monitoring"[tiab] OR "Follow-up"[tiab] OR
"Protocol"[tiab] OR "Guideline"[tiab] OR "Recommendation"[tiab] OR
"Strategy"[tiab] OR "Approach"[tiab] OR "Administration"[tiab] OR "Dose"[tiab]
OR "Dosing"[tiab] OR "Replacement"[tiab] OR "Immunoglobulin"[tiab] OR
"IVIG"[tiab] OR "SCIG"[tiab] OR "IgG"[tiab] OR "Prophylaxis"[tiab] OR
"Antibiotic"[tiab] OR "Antimicrobial"[tiab] OR "Antifungal"[tiab] OR
"Intervention"[tiab] OR "Outcome"[tiab] OR "Complication"[tiab] OR
"Risk"[tiab]
```

Combined: (Population) AND (Pregnancy) AND (Management). Filters: English language; Publication date 2005/04 to 2025/04.

6. Inclusion and Exclusion Criteria

Eligibility criteria applied at title/abstract and full-text screening stages:

- **Inclusion — Population:** Women with a confirmed diagnosis of CVID (per established diagnostic criteria) who were pregnant, planning pregnancy, or had completed pregnancy.
- **Inclusion — Intervention:** Any pregnancy-related management approach, including IgRT (IVIG, SCIG, fSCIG), antimicrobial prophylaxis, structured monitoring, vaccination, or multidisciplinary care.
- **Inclusion — Outcomes:** Reporting of at least one maternal, pregnancy, or fetal/neonatal outcome with patient-level data.
- **Inclusion — Publication:** Full-text articles published in English, 2005–2025.

- **Exclusion:** Abstract-only publications, conference proceedings, editorials, opinion pieces, or narrative reviews without patient-level data; animal or in vitro studies; non-English without accessible translation; articles without CVID-specific or pregnancy-specific data.

7. Records Identified, Screened, and Included

Counts at each stage of the literature screening process. The original Covidence-generated PRISMA-style flow diagram was prepared for the systematic-review framing but was not retained when the manuscript was reframed as a narrative review. The numbers below are reproduced from that Covidence export and constitute the only record of the literature screening flow:

Stage	n
**Records identified — MEDLINE (Ovid)**	236
**Records identified — Embase (Ovid)**	500
**Records identified — hand-search**	0
**Total records before de-duplication**	736
**Duplicates removed (12 manual; 30 by Covidence)**	42
**Records screened (title/abstract)**	694
**Records excluded at title/abstract**	648
**Reports sought for retrieval**	46
**Reports not retrieved**	0
**Full-text reports assessed for eligibility**	46
**Full-text reports excluded — total**	39
**Studies included in narrative synthesis**	7

Note: Five additional full-text records were excluded during the revision from an initial systematic-review framing to the final narrative-review framing, reflecting a tighter focus on studies providing direct CVID-pregnancy clinical-management data with patient-level reporting suitable for the narrative synthesis presented in Table 1 of the main manuscript.

8. Hand-search and Reference-list Review

The reference lists of the most clinically relevant retrieved articles — Mallart et al. 2023 (PREPI), Kralickova et al. 2015, and Zhang & Cunningham-Rundles 2023 — were screened by the lead reviewer for additional eligible studies not captured by the database searches. No additional studies meeting inclusion criteria were identified through this process.

9. Screening Process and Software

All retrieved citations were imported into a structured reference-management workflow for de-duplication, title and abstract screening, full-text review, and data extraction. Title and abstract screening and full-text eligibility assessment were performed independently by three reviewers (F.A., M.A., and G.G.). Each record was independently assessed by at least two reviewers; discrepancies were resolved through discussion or third-reviewer adjudication when required.

10. Included Studies (n = 7)

The seven studies retained for the narrative synthesis are presented in Table 1 of the main manuscript. Full citations and assigned reference numbers (per the main-manuscript reference list):

- **Mallart 2023 [2]** — Mallart, E.; Francoise, U.; Driessen, M.; Blanche, S.; Lortholary, O.; Lefort, A.; Caseris, M.; Fischer, A.; Mahlaoui, N.; Charlier, C.; et al. Pregnancy in primary immunodeficiency diseases: The PREPI study. J. Allergy Clin. Immunol. 2023, 152, 760–770.
- **Manson 2012 [7]** — Manson, A.L.; Zaheri, S.; Kelleher, P.; Wakelin, S.; Nelson-Piercy, C.; Seneviratne, S.L.; Cooper, N. Management of granulomatous common variable immunodeficiency diagnosed in pregnancy: A case report. J. Perinatol. 2012, 32, 387–389.

- **Danieli 2012 [8] —** Danieli, M.G.; Moretti, R.; Pettinari, L.; Gambini, S. Management of a pregnant woman with common variable immunodeficiency and previous reactions to intravenous IgG administration. BMJ Case Rep. 2012, 2012, bcr2012007594.
- **Kralickova 2015 [9] —** Kralickova, P.; Kurecova, B.; Andrys, C.; Krcmova, I.; Jilek, D.; Vlkova, M.; Litzman, J. Pregnancy Outcome in Patients with Common Variable Immunodeficiency. J. Clin. Immunol. 2015, 35, 531–537.
- **Marasco 2017 [10] —** Marasco, C.; Venturelli, A.; Rao, L.; Vacca, A.; Carratù, M.R. Management of common variable immunodeficiency by subcutaneous IgG self-administration during pregnancy — A case report. Clin. Case Rep. 2017, 5, 1309–1311.
- **Sheikhbahaei 2018 [11] —** Sheikhbahaei, S.; Sherkat, R.; Camacho-Ordonez, N.; Khoshnevisan, R.; Kalantari, A.; Salehi, M.; Nazemian, S.S.; Nasr-Esfahani, M.H.; Klein, C. Pregnancy, child bearing and prevention of giving birth to the affected children in patients with primary immunodeficiency disease; a case-series. BMC Pregnancy Childbirth 2018, 18, 299.
- **Egawa 2019 [12] —** Egawa, M.; Kanegane, H.; Imai, K.; Morio, T.; Miyasaka, N. Intravenous immunoglobulin (IVIG) efficiency in women with common variable immunodeficiency (CVID) decreases significantly during pregnancy. J. Matern. Fetal Neonatal Med. 2019, 32, 3092–3096.

The 39 full-text exclusion reasons (categorised at full-text review in Covidence) were as follows. Several records met more than one exclusion criterion; each was counted once toward the total under its primary reason: no management information (study mentions pregnancy but does not discuss management approaches), n = 8; studies on other immunodeficiencies without separately reported CVID data, n = 6; wrong population — studies without confirmed CVID diagnosis, n = 4; no pregnancy data — studies without specific pregnancy data in women with CVID, n = 3; non-English publication without available translation, n = 3; internet surveys relying only on patient-reported CVID diagnosis, n = 2; studies in healthy or pathological pregnancies without CVID patients, n = 2; publications prior to 2005 using different IgG preparations, n = 2; studies on antibody transfer from IVIG-treated mothers without CVID patients, n = 2; insufficient outcome data — pregnancy mentioned but no outcomes reported, n = 2; wrong study type — conference abstract, editorial, or opinion without patient data, n = 2; not relevant to the research question, n = 2; full text unavailable after reasonable attempts, n = 2; and quality-improvement analysis of PID women's perceptions in pregnancy without medical-management data, n = 1.

The seven included studies were further categorised by topic (per the original screening record): general CVID-pregnancy cohort studies (n = 2; Mallart 2023, Kralickova 2015), SCIG in pregnancy (n = 1; Marasco 2017), and IVIG-focused case reports and series (n = 4; Manson 2012, Danieli 2012, Sheikhbahaei 2018, Egawa 2019).
