# Supplementary material for: Common Variable Immune Deficiency and Pregnancy: Improving Outcomes Through Multidisciplinary Care
Source: J Clin Med. 2026 May 15;15(10):3810. doi: 10.3390/jcm15103810 (PMC13206869; doi:10.3390/jcm15103810)
Supplement: Supplementary file 1 [file jcm-15-03810-s001.zip › File_S2_QualityAssessment.pdf]

Supplementary File S2: Methodological Quality Summary of Included Studies

Manuscript: Common Variable Immune Deficiency and Pregnancy: Improving Outcomes Through Multidisciplinary Care.

Article type: Narrative review with structured literature search.

Purpose of this supplement: This document is provided for transparency — narrative reviews do not require formal risk-of-bias scoring. It is included as a structured summary of the design, sample size, comparator presence, key methodological strengths and limitations, and overall narrative evidence-strength judgement for each included study, to help the reader weight the findings discussed in the main text.

1. Methodology

The methodological quality of each of the seven studies included in the narrative synthesis (presented in Table 1 of the main manuscript) was appraised independently by three reviewers (F.A., M.A., and G.G.). The Joanna Briggs Institute (JBI) Critical Appraisal Checklists for Case Reports and Case Series were used as a structured guide to inform reviewer judgement on dimensions including clarity of patient and intervention reporting, demographic and clinical characteristics, completeness of outcome ascertainment, follow-up adequacy, and applicability of conclusions.

A formal numerical risk-of-bias instrument (such as the Newcastle–Ottawa Scale [23]) was not applied, given the predominance of single-patient case reports and small retrospective case series, the absence of randomised or controlled comparator designs, and the narrative-review framing of this synthesis. This decision is stated explicitly in the Limitations of the main manuscript.

Each study was assigned an overall evidence-strength rating (Strong / Moderate / Limited) by reviewer consensus, reflecting the cumulative weight of: study design, sample size, presence and adequacy of a comparator group, completeness of outcome reporting, and applicability to the clinical question. Disagreements between reviewers were resolved through discussion. The contemporary single-centre cohort by Kalkan et al. (2026; n = 33), cited in the main manuscript Discussion, is treated as contextual evidence and was not part of the seven studies appraised in this supplement.

2. Rating Definitions

- **Strong:** Multicentric or larger single-centre retrospective cohort with explicit comparator data and standardised outcome capture. The strongest evidence available in this rare-disease context, though still observational.
- **Moderate:** Retrospective case series with structured within-cohort reporting but without an external comparator group; small sample size limits generalisability.
- **Limited:** Single-patient case reports. Informative for unusual presentations or novel interventions but contribute weakly to generalisable management recommendations.

3. Evidence-Strength Summary Table

#	Study [Ref]	Design	n (patients / pregnancies)	Country	Comparator	Strength

**1**	Mallart 2023 — PREPI [2]	Retrospective observational, multicentric	51 / 119 (82 live births, 27 miscarriages, 2 ectopic, 7 voluntary terminations, 1 medical termination)	France	Yes — IgRT-replete vs. suboptimal; risk-factor analysis (history of severe infection)	Strong
**2**	Kralickova 2015 [9]	Retrospective cohort	50 / 115	Czech Republic	Yes — vs. Czech general-population reference data	Strong
**3**	Egawa 2019 [12]	Retrospective case series	4 / 9 (8 live births, 1 miscarriage)	Japan	None	Moderate
**4**	Sheikhbahaei 2018 [11]	Retrospective case series (CVID subset within PID series)	3 CVID / within 9-PID cohort	Iran	None	Moderate
**5**	Manson 2012 [7]	Single case report	1 / 2 (2 live births)	United Kingdom	N/A	Limited
**6**	Danieli 2012 [8]	Single case report	1 / 2 (1 live birth, 1 miscarriage)	Italy	N/A	Limited
**7**	Marasco 2017 [10]	Single case report	1 / 2 (2 live births)	Italy	N/A	Limited

4. Per-Study Appraisal

Mallart et al. 2023 — PREPI [2] — *Evidence strength: Strong*

Strengths: Largest multicentric primary-antibody-deficiency pregnancy cohort to date; standardised cross-centre data capture; comprehensive maternal, obstetric, and neonatal outcome reporting; identifies risk factors (history of severe infection) for adverse outcomes; published in a high-impact specialty journal.

Limitations: Retrospective design with attendant ascertainment and selection bias; broad data-collection window (1966–2022) spanning major changes in IgRT practice; heterogeneous PAD diagnoses (not exclusively CVID); no non-PAD comparison group; IgRT product not always specified; possible centre-level practice variation.

Kralickova et al. 2015 [9] — *Evidence strength: Strong*

Strengths: One of the larger single-country CVID pregnancy series; structured comparison of pre-CVID, pre-IgRT, and on-IgRT pregnancies; comparison to national Czech population data offers an external reference; outcomes reported across the full obstetric and neonatal spectrum (preterm labour, eclampsia, low birthweight, stillbirth).

Limitations: Retrospective; no individual-level comparator group beyond population reference rates; IgRT product not always specified; heterogeneous subgroups within a small overall cohort; potential ascertainment bias from referral patterns; study years not always explicit.

Egawa et al. 2019 [12] — *Evidence strength: Moderate*

Strengths: Quantitative trimester-specific IVIG efficiency data — one of the few mechanistic pharmacokinetic observations in CVID pregnancy; IgG trough levels reported per trimester; defined study period (January 2007 – December 2016); clear description of dose adjustments.

Limitations: Small n (4 patients, 9 pregnancies); single-centre retrospective design; no comparator group; heterogeneous IgRT regimens (IVIG with one SCIG case); limited statistical power; outcome ascertainment limited to one-month neonatal follow-up.

Sheikhbahaei et al. 2018 [11] — *Evidence strength: Moderate*

Strengths: Reports outcomes from a primary-immunodeficiency centre in a geographic region underrepresented in the CVID-pregnancy literature; describes IgRT-naïve vs. IgRT-treated pregnancies and includes informative individual narratives.

Limitations: CVID patients form only a small subset (n = 3) within a broader nine-patient PID cohort; retrospective; no comparator; IgRT product and dose details inconsistently reported; small n limits external validity; reporting bias possible given case-series design.

Manson et al. 2012 [7] — *Evidence strength: Limited*

Strengths: Detailed individual-patient narrative; clearly reported IgRT regimen with trimester-based dose escalation; complete reporting of maternal complications (deep vein thrombosis, pseudomonas bacteraemia, cholestasis, placental abruption, post-partum haemorrhage) and neonatal outcome; informative on granulomatous CVID first manifesting in pregnancy.

Limitations: n = 1; no comparator; selection and reporting bias; limited generalisability; IVIG product not specified; uncertain how representative the clinical course is of granulomatous-CVID pregnancies generally.

Danieli et al. 2012 [8] — *Evidence strength: Limited*

Strengths: Detailed narrative; IgRT product and dose explicit; informative description of managing IVIG infusion reactions and the substitution to a 10% liquid preparation (Privigen®) in pregnancy; useful for clinicians facing CVID patients with IVIG intolerance.

Limitations: n = 1; no comparator; selection and reporting bias; outcome data limited to a single pregnancy course on the alternative regimen; first pregnancy ended in miscarriage at 8 weeks, limiting outcome data per pregnancy.

Marasco et al. 2017 [10] — *Evidence strength: Limited*

Strengths: Detailed narrative; SCIG product, dose, and third-trimester dose escalation explicit; informative on SCIG self-administration during pregnancy and on IgRT initiation post-CVID-diagnosis in a multiparous patient; useful complement to IVIG-focused cases.

Limitations: n = 1; no comparator; uncomplicated obstetric course limits inference about adverse-event risk on SCIG in pregnancy; outcomes reported only to delivery.

5. Overall Synthesis Statement

Strong evidence (n = 2): Mallart 2023 (PREPI) and Kralickova 2015 contribute comparator data and represent the highest-quality evidence in the synthesis. Both are retrospective in design, but PREPI is the largest multicentric primary-antibody-deficiency pregnancy cohort to date, and Kralickova provides a single-country cohort of 50 CVID patients with general-population reference data. The recently published single-centre cohort of Kalkan et al. (2026; n = 33), cited in the main manuscript Discussion, is contemporary contextual data and is not part of the seven studies appraised here.

Moderate evidence (n = 2): Egawa 2019 and Sheikhbahaei 2018 are small retrospective case series providing detailed within-cohort observations on IgRT dosing and obstetric outcomes, but are limited by absence of comparator groups and small sample size.

Limited evidence (n = 3): Manson 2012, Danieli 2012, and Marasco 2017 are single case reports — each informative for unusual presentations or novel management approaches (granulomatous CVID first manifesting in pregnancy; alternative IVIG product after infusion reactions; SCIG self-administration during pregnancy) but contributing weakly to generalisable claims.

6. Implications for the Narrative Synthesis

The predominance of case-report and small-case-series evidence is the principal reason this article is presented as a narrative review rather than as a systematic review with quantitative synthesis. Throughout the main manuscript, findings derived from the cohort studies (Mallart 2023 and Kralickova 2015) are weighted more heavily and named explicitly, while findings from the case series (Egawa 2019; Sheikhbahaei 2018) and the individual case reports (Manson 2012; Danieli 2012; Marasco 2017) are flagged as illustrative rather than confirmatory.

Recommendations in the proposed management algorithm are explicitly labelled as consensus- and experience-based unless directly supported by cohort-level data, in which case the contributing study is cited in-line. Prospective multicenter studies and international primary-immunodeficiency registries are needed to elevate the underlying evidence base for clinical decision-making in this rare and clinically heterogeneous patient population.
